# The surgical treatment of Parsonage-Turner Syndrome: A PRISMA scoping review

**DOI:** 10.1007/s10143-025-04013-y

**Published:** 2026-01-03

**Authors:** Michael J. Albdewi, Alex Clayton Donaghy, Yoav Morag, Amro Stino, Benjamin Becker, Noojan J. Kazemi, Yamaan S. Saadeh

**Affiliations:** 1 University of Michigan Medical School, Ann Arbor, MI USA; 2 Department of Physical Medicine and Rehabilitation, University of Michigan, Ann Arbor, MI USA; 3 Department of Radiology, University of Michigan, Ann Arbor, MI USA; 4 Department of Neurology, University of Michigan, Ann Arbor, MI USA; 5 Department of Neurosurgery, University of Michigan, Ann Arbor, MI USA

**Keywords:** Brachial plexitis, Neuralgic amyotrophy, Parsonage-Turner Syndrome, Surgical intervention, Scoping review

## Abstract

Parsonage-Turner Syndrome (PTS) is characterized by an abrupt onset of severe shoulder and upper arm pain followed by motor weakness, muscle atrophy, and sensory disturbances. Although spontaneous recovery is possible, many patients experience prolonged disability. Recent advances in imaging have facilitated the identification of surgically treatable nerve constrictions, prompting interest in surgical interventions including interfascicular neurolysis, decompression, and nerve transfers. This study aims to assess the efficacy of surgical treatment for PTS, delineate optimal patient selection and timing, and compare surgical outcomes with conservative management approaches. A scoping review, adhering to PRISMA guidelines, was conducted using PubMed and Embase databases. Two independent reviewers screened 1,296 articles, with 4 retrospective cohort studies, 1 case series, and 1 case report meeting inclusion criteria. Sample sizes ranged from 1 to 59, and studies were published between 2011 and 2023. Data extracted included study design, sample size, intervention type, outcome measures, and timing of intervention. Results demonstrated that surgical treatment was associated with significant improvements in muscle strength and sensory outcomes. In comparative analyses, patients undergoing surgery exhibited superior recovery relative to those managed conservatively, particularly among younger individuals and those with shorter symptom durations. The average time to surgical intervention was approximately 11 months after symptom onset, with evidence suggesting that earlier surgical management may enhance clinical recovery. This preliminary data suggests that, in carefully selected patients, surgical intervention may have a potential benefit for patients with PTS, particularly for those who fail to respond to conservative management and present early in their disease course. Despite promising outcomes, the limited quality, small sample size, retrospective nature and heterogeneity of the current literature highlight the need for prospective, controlled trials to establish standardized treatment protocols and optimize patient selection.

**Supplementary Information:**

The online version contains supplementary material available at 10.1007/s10143-025-04013-y.

## Introduction

Parsonage-Turner Syndrome (PTS), also known as brachial plexitis or neuralgic amyotrophy, is a disorder encompassing a broad spectrum of presentations, usually beginning with acute onset of asymmetric, upper extremity pain followed by neurological deficits such as motor weakness, muscle atrophy, allodynia, and dysesthesias after a few days [1]. The exact etiology of PTS remains unclear but it is believed to involve immune-mediated or vasculitic mechanisms targeting the brachial plexus and/or individual nerves due to mechanical susceptibility and/or compromise in the epineural blood-nerve barrier, often manifesting in the setting of preceding infection, surgery, trauma, or vaccination [1–4].

**Fig. 1:**
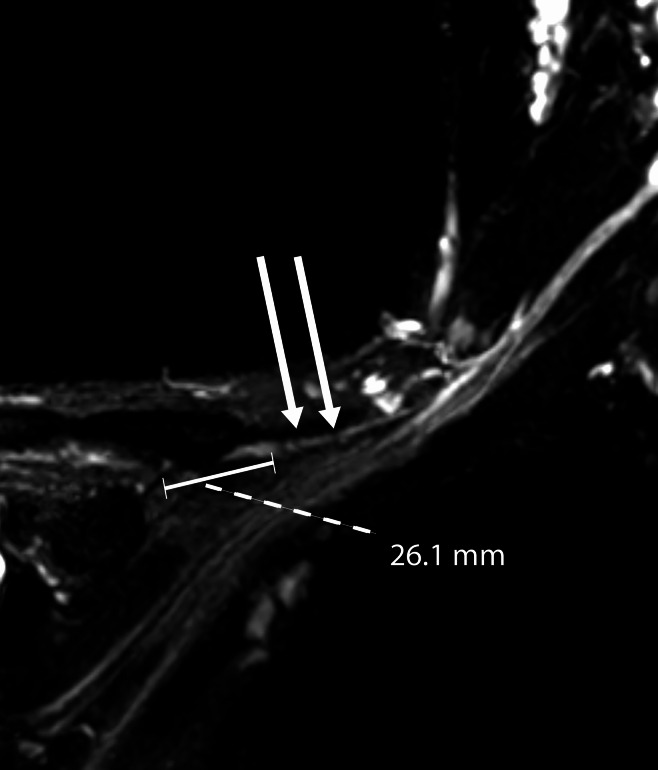
Coronal 3D SPACE sequence depicts 2 areas of focal hourglass constrictions (arrows) involving the suprascapular nerve, located approximately 2.6 cm medial to the suprascapular notch area

PTS detection has significantly increased, with an annual incidence of 1 per 1,000 individuals [5]. While the diagnosis of PTS is largely clinical, electrodiagnostic studies (EDx) are essential for diagnostic confirmation and to exclude disease mimickers [1]. Isolated involvement of proximal non-appendicular motor nerves (e.g., the suprascapular nerve) should prompt a targeted clinical and Edx evaluation, as appendicular upper limb Edx testing may be normal [6]. Imaging studies have also proven highly useful, which may reveal intramuscular changes within select peripheral nerve distributions, focal hourglass-type constrictions (HGCs), and increased signal on T2 weighted sequences (Fig. 1) of the involved nerve segments [7, 8]. High-resolution ultrasound may help localize focal, characteristic lesions including HGC, which may inform decisions to proceed with neurolytic surgery [9]. The role of imaging in PTS has been revolutionary, increasing the identification of potential targets for surgical intervention (SI).

The natural history of PTS involves spontaneous recovery with long-term sequelae and resolution still being studied [1]. The current literature suggests poor outcomes in patients with PTS, for example, in a large cohort study of over 200 patients, less than 10% achieved full recovery, and nearly 25% were unable to return to work 3 years after the onset of PTS [10]. Furthermore, another study displayed over 25% of patients experiencing pain and fatigue 2 years post-diagnosis and over 50% of patients needing to change their profession or retire from work prematurely [11].

Interventions for PTS include both conservative and surgical strategies. Conservative treatments include pain management, corticosteroids, electrical nerve stimulation, and physical/occupational therapy [1]. Conservative strategies alone rely on enhancing natural recovery by maximizing available muscular strength and function, but do not directly address the underlying pathology. This contrasts with SIs that may involve decompression, neurolysis, and/or nerve transfers [1, 12, 13]. Decompression targets any nerve constriction impeding nerve function and recovery. Neurolysis targets hourglass-like or fascicular constrictions found on imaging to relieve nerve compression and facilitate nerve regeneration [13]. Nerve transfers involve the surgical transfer of a healthy donor nerve to an affected nerve to restore function [12]. Given limited data on the optimization of SI for PTS, we wanted to develop an overview of the existing literature on the topic and summarize the quality and type of emerging evidence for this potential treatment. In addition to these aims, due to the lack of large-cohort studies on this topic, we undertook a scoping review to evaluate existing literature pertaining to the clinical outcomes of SI for PTS, identify optimal candidates and timing for surgery, and compare the effectiveness of surgical versus conservative treatment approaches.

## Materials and methods

**Fig. 2:**
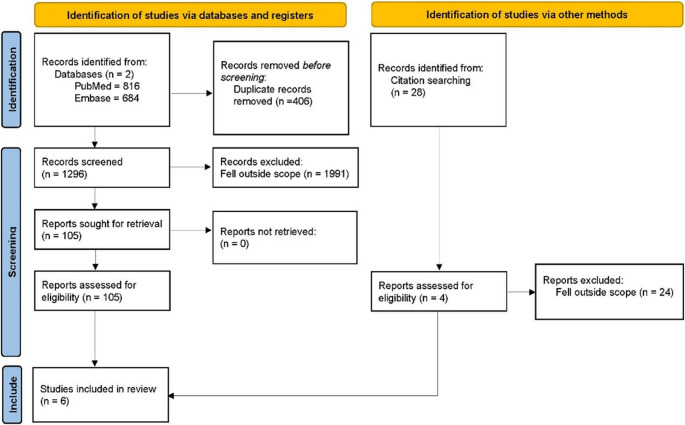
PRISMA flow diagram

Using PRISMA guidelines for scoping reviews [14] (Fig. 2), systematic searches of PubMed and Embase databases were performed by two independent reviewers to identify studies reporting the surgical treatment of PTS. Inclusion criteria included peer-reviewed journal publication, human participants who were diagnosed with PTS or a brachial plexopathy, patients receiving surgical treatment, and in English. Eligibility included quantitative, qualitative, and mixed methods to consider different interventions. Studies were excluded if they were not peer reviewed, did not include patients with PTS or brachial myelopathy, were not published, or were not in English. We limited our search to English-language studies due to resource constraints, including the unavailability of reliable translation services, which could introduce inaccuracies and bias. While this approach may risk omitting relevant findings published in other languages, it ensures consistency in data interpretation and maintains the methodological rigor of our analysis. No limit was set on the publication date.

The main search strategy was created in PubMed and translated to Embase. Three sentinel articles were used to harvest Medical Subject Heading (MeSH) terms. The search was then constructed by combining harvested keywords and MeSH terms for variance in treatment choice and outcomes in patients with PTS in PubMed: (“Brachial Plexus Neuritis”[Mesh] OR “Brachial Plexus Neuropathies”[Mesh] OR “Parsonage Turner Syndrome”[Title/Abstract]) AND (“Surgery”[Subheading] OR “Nerve Transfer”[Mesh] OR “Decompression, Surgical”[Mesh] OR “Spinal Fusion”[Mesh]) AND “Humans”[Mesh] AND (“Treatment Outcome”[Mesh] OR “Postoperative Complications”[Mesh] OR “Recovery of Function”[Mesh] OR “Follow-Up Studies”[Mesh]).

To ensure a comprehensive search, a similar search was conducted using harvested keywords and MeSH terms in Embase: (‘brachial plexus neuritis’/exp OR ‘brachial plexus neuropathy’/exp OR “Parsonage Turner Syndrome”:ti, ab) AND (‘surgery’/exp OR ‘nerve transfer’/exp OR ‘surgical decompression’/exp OR ‘spinal fusion’/exp) AND ‘human’/de AND (‘treatment outcome’/exp OR ‘postoperative complication’/exp OR ‘function recovery’/exp OR ‘follow up’/exp). All searches were completed by November 2024. A full description of the search strategy and a complete list of search terms and limits used in both databases is included in the Supplementary Information. The review protocol was submitted to PROSPERO (ID: 1069876).

Two independent reviewers screened 816 publications from PubMed and 684 publications from Embase. There were 406 duplicates leaving 1,296 articles to screen. Reviewers independently evaluated titles, abstracts, and full texts to identify relevant publications based on the inclusion and exclusion criteria. Rayyan software was used for article refining and duplicate screening. A data charting form was developed to determine variables to extract. One reviewer independently charted the data and updated the data-charting form as appropriate.

Publications were reviewed and the following data were extracted from each study: (1) study type, (2) sample size, (3) presenting diagnoses, (4) treatment groups, (5) SIs, (6) outcomes, and (7) notable limitations. The Newcastle-Ottawa Scale (NOS) was used for critical appraisal of the individual sources of this review; charting of NOS results for each study are provided (See Supplementary Information 2) [15]. As all studies obtained a NOS score over 6, further interpretation of the studies was conducted understanding the low risk of bias present. Studies were summarized by experimental design and thematic findings. Systematic reviews that fell under our search were examined for any studies that were not initially identified in our search; four studies were identified in this manner.

## Results

Two databases (PubMed, Embase) were systematically searched by 2 reviewers for publications related to the surgical treatment of PTS. Based on 3 sentinel articles, relevant search terms were derived, which resulted in a total of 816 articles from PubMed and 684 articles from Embase. Following the initial search, 105 publications were identified that met the inclusion criteria based on title and abstract. Upon further reading of the manuscripts, 22 of these articles met inclusion criteria. Specifically, 8 articles were identified from PubMed and 14 from Embase (Fig. 2). However, of these, 2 articles from PubMed and 1 from Embase were not original articles, 1 publication from PubMed and 2 from Embase were not written in English, 1 publication from Embase was an abstract without an available full-text document, 1 publication was unable to be extracted, and 6 publications from PubMed and 9 from Embase were not related to PTS upon reading. Examining systematic reviews that fell under our search identified four relevant articles. This resulted in 6 distinct publications included in our review. Four were retrospective studies [16–19], 1 was a case series [12], and 1 was a case report [20]. Publication dates ranged from 2011 to 2023 (Table 1). Two of the articles met a NOS score of 7/9 [16, 18] with 2 having a NOS of 6/9 [17, 19].

**Table 1:** Study characteristics

Source	Sample size	Sex distribution	Average age (+/- STD)	Treatment groups	Surgical technique
Krishnan et al., 2021 [16]	24	Not specified	48.5 +/- NA 50 for surgical patients, 47 for nonsurgical patients	Surgical and conservative (physical therapy, with one patient receiving a corticosteroid injection)	Microsurgical epi- and peri-neurolysis of HGCs
Akane et al., 2016 [17]	59	19 F, 32 M	44.1 +/- NA	Surgical and conservative (unspecified)	Neurolysis
Pan et al., 2011 [18]	5	2 F, 3 M	31.8 +/- 9.6	Surgical and conservative (physiotherapy, vitamin B12 supplementation)	Neurolysis, nerve graft, neurorrhaphy
Lenkeit et al., 2023 [20]	1	1 M	16	Surgical and conservative (steroids, intensive physiotherapy)	Nerve transfer
Winter et al., 2022 [12]	4	1 F, 3 M	54	Surgical	Neurolysis, nerve transfer
Solaja et al., 2023 [19]	10	1 F, 9 M	51.3 +/- 9.7	Surgical	Neurolysis, nerve transfer

### Study characteristics

Study sample sizes ranged from 1 to 59 participants. All studies except for 2 [12, 19] specified both nonsurgical and surgical groups [16–18], Muscle strength was the most commonly assessed clinical outcome, having been evaluated in all studies [12, 16–19]. Three studies examined the progression of sensory symptoms [12, 17, 18]. One study assessed improvements in Hand20 score [17]. The average age of the participants in the studies was 40.2 years (IQR 34.9–50.6) years old. Roughly 22.8% (IQR 10-38.8%) of participants in each study were females. Diagnostically, 1 study confirmed hourglass-like fascicular constrictions (HLFC) with ultrasound [16]. Three studies used MRI for confirmation of nerve compression sites [12, 17, 20]. And 3 studies used EMG to confirm muscle deterioration [12, 18, 20] followed by surgical exploration of potential compressive sites in one [18].

### Interventions

In the studies, 76.6% (IQR 45.8–100%) of patients underwent SIs. All studies implemented interfascicular neurolysis as the SI [12, 16–20]. Three studies included physical therapy as a conservative treatment option [16, 18, 20], and 2 studies utilized steroid injections [16, 20]. A single study utilized vitamin B_12_ supplementation as conservative treatment [18].

### Indications and timing of SI

All the studies provided indications for SI, all related to a lack of improvement with nonsurgical management based on the clinical scoring tool used (Table 2). One study defined failure to improve as the inability to achieve improvements based on MRC grades [18]. The average time to SI after the onset of PTS was 11.2 months (IQR 10.6–12.8). Only 1 study explained the delay, attributing it to the patient’s referral from another facility [16].

**Table 2:** Summary data on surgical indications, follow-up timeline, and study outcomes

Source	Indications for surgery	Average follow-up duration	Outcomes
Krishnan et al., 2021 [16]	1. Lack of recovery at greater than 12 months of PTS onset with evidence of significant denervation OR 2. No clinical recovery after 6 months of PTS onset verified by 3 exams, also testing for complete denervation	From surgical treatment: 14.8 months From PTS onset (surgically treated patients): 27.3 months	• Recovery of at least M3 strength was observed in 12/15 nerves in 81.8% of surgically treated patients • Recovery of at least M4 strength was achieved in 10 nerves in 72.7% of surgically treated patients • 4/16 nerves in 23.1% of conservatively treated patients experienced at least M4 recovery • 20% of conservatively treated patients with radial nerve palsy recovered • MRC recovery was significantly greater among the surgically treated patients
Akane et al., 2016 [17]	Lack of “recovery signs” at greater than 3 months of PTS onset	From PTS onset: 21.7 months	• 72% of patients treated with neurolysis experienced motor function recovery within 3 months, • 71.4% of conservatively treated patients experienced recovery within 5.4 months • Neurolysis improved Hand20 scores compared to conservative treatment, but did not meet statistical significance
Pan et al., 2011 [18]	Failure to improve based on clinical symptoms and EMG measurements after 2 to 11 months of conservative treatment	From PTS onset: 48 months	• Recovery to at least MRC grade 4 was achieved in all nerves treated with neurolysis • 40% of nerves treated with resection, neurorrhaphy, or nerve grafts experienced full recovery • All pain symptoms resolved with surgery
Lenkeit et al., 2023 [20]	Failure to improve based on neurologic analysis and clinical exam 12 months after symptom onset	From surgery: 12 months	• Improvement to MRC grade 4/5 and ROM of 130 degrees from pre-op MRC grade 2/5 and ROM of 90 degrees
Winter et al., 2022 [12]	1. No motor units present on EMG 5 months after symptom onset OR 2. Patient presentation included paresis for > 5 months after symptom onset	From surgery: 8.25 months	• Patient A: MRC improvements of 0 and 3/5 -> 5/5 • Patient B: MRC improvements of 0 and 3/5 -> 5/5
Solaja et al., 2023 [19]	Patients without “electrodiagnostic evidence” of improvement after 5 months of symptom onset	From surgery: 14.8 +/- 3.2 months	• MRC improvement from a median of 1.5 to 4 • 90% of patients achieved an MRC grade ≥ 4 • Electrodiagnostic follow-up was available for nine patients, 8/9 demonstrated increased motor units and improved recruitment postoperatively

### Notable findings

All studies evaluating muscle strength reported significant improvements following SI [12, 16–20]. Only 1 compared surgical to conservative treatment and demonstrated that surgically treated patients had a significantly greater recovery of muscle strength compared to those receiving conservative treatment [16]. One study reported improved nonmotor clinical outcomes for surgically treated patients compared to those treated conservatively [16]. All studies evaluating the progression of sensory symptoms reported improvements in postoperative sensation, with one study highlighting decreases in paresthesia, dysesthesia, and allodynia [12, 17, 18].

## Discussion

This review identified 6 articles evaluating the surgical treatment of PTS [12, 16–20]. Most studies assessed muscle strength using different subjective scoring systems and reported clinical improvements associated with SI, most notably in younger patients and those with shorter symptom duration.

The types of interventions varied, but all had a surgically treated cohort [12, 19, 20], with 3 having an additional nonsurgically treated cohort [16–18]. SI included interfascicular neurolysis of HGCs [12, 16, 17, 19, 20] and end-to-end nerve transfer [12, 18–20], with 1 study having an additional neurorrhaphy and autografting [18]. Conservative management strategies included physical therapy [16, 18, 20], corticosteroid injections [16], and vitamin B12 supplementation [18]. A common limitation of most studies included a small sample size [12, 16, 19, 20] and a nonrandomized retrospective study without a control group [16, 19].

Improvements in strength and sensation were noted in all studies regardless of treatment strategy. When comparing surgical and conservative interventions, many studies noted greater clinical improvements in the surgically treated cohorts. One study found an improved MRC strength to at least M3 in 81.8% and M4 in 72.7% of surgically treated patients compared to 23.1% of conservatively treated patients attaining at least M4 [16]. It was noted that muscles that failed to recover, despite surgical or conservative treatment, were most often radial-innervated [16]. It is unclear why the radial nerve is associated with poorer healing; however, it may be due to a greater number of HGCs being present on the radial nerve [16].

Earlier timing of surgical management may be associated with improvements in clinical recovery [16]. In a review article on the surgical treatment of neuralgic amyotrophy, Pöschl et al. [21] highlighted that earlier SI led to improved outcomes, but that there remains heterogeneity in surgical approach and surgical procedure indicated. All studies in our review used a failed response to conservative management as the indication for surgical management, with different time criteria and measurement tools to measure failure. The average time to surgical intervention after the onset of PTS was 13.1 months (IQR 12.8–13.3), with delays in patient referrals contributed to delayed management [16]. Hagemann et al. [22] recommended waiting for improvement following 3 months of conservative treatment before pursuing further diagnostic imaging to identify potential SI sites. No study compared time to initial recovery between conservatively managed and surgically managed groups. Krishnan et al. [16] noted an average time to initial improvement of 4.19 months postoperatively. As with most peripheral nerve surgeries, earlier intervention is beneficial; however, this must be weighed against the possibility of spontaneous recovery. Published rates of spontaneous resolution are heterogenous and range from 10% to 90% [10, 23]. This uncertainty reflects the need for a better understanding of prognostic factors predicting recovery in patients with PTS, and prospective controlled trials critically evaluating the efficacy of surgical and non-surgical treatments for PTS. This type of study would allow for a much better assessment of the true effectiveness of surgery in this condition, while elucidating a better understanding of a potential placebo effect. Electromyographic, imaging, or clinical exam changes at various time intervals early in the disease course may offer insight, but clear guidelines as to “who and when” have not been established. It is important to note prognostic factors affecting the likelihood of spontaneous recovery to offer timely SI to patients needing it.

There is a lack of high-quality evidence, controlled trials, comparing the efficacy of surgical versus conservative management for PTS. Existing studies are primarily retrospective and therefore are limited in the strength of their conclusions. In surgically treated patients, it remains unclear whether observed clinical benefits are due to the natural course of PTS recovery, the surgical intervention itself, or a potential surgical placebo effect. However, the presented literature suggests that earlier surgical treatment in patients with intervenable targets based on imaging findings could result in improved strength recovery compared to non-surgical intervention. The heterogeneity in study design and reported outcomes limits the comparison of the presented studies, and the limitations of the studies restricts clear clinical recommendations from being established. This underscores the need for prospective studies to better understand the indications for the surgical management of PTS and to determine which specific procedures are most appropriate for individual patients.

### Limitations

This scoping review has several limitations, primarily due to the retrospective nature of all included studies, which introduces potential biases related to data collection, recall accuracy, and limits the ability to establish causality or rigorous evidence-based conclusions. The lack of consistently reported patient-reported outcome measures is a significant limitation of this review. Excluding non-English studies increases the risk of language bias. Relying on the different subjective assessments of each study to determine failure of conservative treatments limits the comparability and standardization of clinical decision-making in similar settings. None of the studies were randomized controlled trials, and the sample sizes of the included studies were not large. The variability in study design, sample sizes, follow-up periods, and the quality and completeness of reported data also affect the reliability of the overall analysis, underscoring the need for future well-designed controlled trials to better assess the interventions.

## Conclusion

This review reported clinical improvements following surgical intervention for PTS of the brachial plexus, particularly in terms of measures of motor strength and patient reported sensory symptoms. Younger patients and those with shorter symptom duration may experience increased benefits from surgery, underscoring the importance of timely intervention. While preliminary data of surgical treatments such as interfascicular neurolysis, decompression, and end-to-end nerve transfers showed clinical improvement, patients experience positive outcomes after conservative treatments as well. The heterogeneity in treatment approaches, the small sample sizes across studies, the lack of consistent patient-record outcome measures, and the retrospective nature of the studies indicate the need for more rigorous, large-scale, controlled trials with well-accepted diagnostic and therapeutic criteria to establish standardized treatment protocols. Future research should focus on better characterization of the natural history of PTS with spontaneous recovery rates to contextualize surgical outcomes, identifying specific patient subgroups that would most benefit from surgical versus conservative management, and delineate factors impacting likelihood of spontaneous recovery, thereby optimizing individual patient outcomes. Overall, this preliminary data suggests that surgical intervention may potentially offer a viable and effective treatment option for PTS.

## Supplementary Information

Below is the link to the electronic supplementary material.

S1. A full description of the search strategy and a complete list of search terms and limits used in both databases. (13.7 KB)

S2. Completed critical appraisal system checklist. (99.3 KB)

## Data Availability

No datasets were generated or analysed during the current study.

## Declarations

### Previous presentation

N/A.

### Previous publication

N/A.
