# Supplementary material for: The surgical treatment of Parsonage-Turner Syndrome: A PRISMA scoping review
Source: Neurosurg Rev. 2026 Jan 3;49(1):97. doi: 10.1007/s10143-025-04013-y (PMC12764620; doi:10.1007/s10143-025-04013-y)
Supplement: Supplementary file 1 — S1. A full description of the search strategy and a complete list of search terms and limits used in both databases. (13.7 KB) [file 10143_2025_4013_MOESM1_ESM.docx]

**Supplementary Information, Full Search Strategy**

***Sentinel Articles***

Winter J, Karir A, Clark TA, Giuffre JL. Surgical treatment of parsonage turner syndrome with primary nerve transfers: a case series and cadaver dissection. *Ann Plast Surg*. 2022;89(3):301-305. doi:10.1097/SAP.0000000000003265. PMID: 35993685

***Search terms collected:*** Brachial Plexus Neuritis*, Brachial Plexus*, Cadaver, Forearm, Humans, Nerve Transfer*, Peripheral Nervous System Diseases*

Lubelski D, Pennington Z, Kopparapu S, et al. Nerve transfers after cervical spine surgery: multi-institutional case series and review of the literature. *World Neurosurg.* 2021;156:e222-e228. doi:10.1016/j.wneu.2021.09.039. PMID: 34536618

***Search terms collected:*** Accessory Nerve, Aged, Brachial Plexus Neuropathies, Cervical Vertebrae, Cohort Studies, Decompression, Female, Follow-Up Studies, Humans, Male, Middle Aged, Nerve Transfer, Postoperative Complications, Postoperative Complications, Range of Motion, Articular, Recovery of Function, Retrospective Studies, Spinal Fusion, Treatment Outcome

Pöschl P, Pham M, Pedro MT, Antoniadis G. Neuralgic amyotrophy: an inflammatory neuropathy and its surgical treatment]. Handchir Mikrochir Plast Chir. 2024;56(1):40-48. doi:10.1055/a-2226-4260. PMID: 38272037

***Search terms collected:*** Brachial Plexus Neuritis*, Brachial Plexus Neuritis*, Brachial Plexus*, Humans, Median Nerve, Peripheral Nerves, Retrospective Studies

**Searches**

**PubMed**

***Final Search***

("Brachial Plexus Neuritis"[Mesh] OR "Brachial Plexus Neuropathies"[Mesh] OR "Parsonage Turner Syndrome"[Title/Abstract]) AND ("Surgery"[Subheading] OR "Nerve Transfer"[Mesh] OR "Decompression, Surgical"[Mesh] OR "Spinal Fusion"[Mesh]) AND "Humans"[Mesh]

AND ("Treatment Outcome"[Mesh] OR "Postoperative Complications"[Mesh] OR "Recovery of Function"[Mesh] OR "Follow-Up Studies"[Mesh]), **N = 816**

**Embase**

***Final Search***

('brachial plexus neuritis'/exp OR 'brachial plexus neuropathy'/exp OR "Parsonage Turner Syndrome":ti,ab) AND ('surgery'/exp OR 'nerve transfer'/exp OR 'surgical decompression'/exp OR 'spinal fusion'/exp) AND 'human'/de AND ('treatment outcome'/exp OR 'postoperative complication'/exp OR 'function recovery'/exp OR 'follow up'/exp), **N = 684**
