# Supplementary material for: The surgical treatment of Parsonage-Turner Syndrome: A PRISMA scoping review
Source: Neurosurg Rev. 2026 Jan 3;49(1):97. doi: 10.1007/s10143-025-04013-y (PMC12764620; doi:10.1007/s10143-025-04013-y)
Supplement: Supplementary file 2 — S2. Completed critical appraisal system checklist. (99.3 KB) [file 10143_2025_4013_MOESM2_ESM.pdf]

NEWCASTLE - OTTAWA QUALITY ASSESSMENT SCALE
COHORT STUDIES

Note: A study can be awarded a maximum of one star for each numbered item within the Selection and Outcome categories. A maximum of two stars can be given for Comparability

Selection

1) Representativeness of the exposed cohort
   a) truly representative of the average patient with PTS (describe) in the community ✵
   b) somewhat representative of the average _______________ in the community ✵
   c) selected group of users eg nurses, volunteers
   d) no description of the derivation of the cohort

2) Selection of the non exposed cohort
   a) drawn from the same community as the exposed cohort ✵
   b) drawn from a different source
   c) no description of the derivation of the non exposed cohort

3) Ascertainment of exposure
   a) secure record (eg surgical records) ✵
   b) structured interview ✵
   c) written self report
   d) no description

4) Demonstration that outcome of interest was not present at start of study
   a) yes ✵
   b) no

Comparability

1) Comparability of cohorts on the basis of the design or analysis
   a) study controls for ______________ (select the most important factor) ✵
   b) study controls for any additional factor ✵ (This criteria could be modified to indicate specific control for a second important factor.)

Outcome

1) Assessment of outcome
   a) independent blind assessment ✵
   b) record linkage ✵
   c) self report
   d) no description

2) Was follow-up long enough for outcomes to occur
   a) yes (select an adequate follow up period for outcome of interest) ✵
   b) no

3) Adequacy of follow up of cohorts
   a) complete follow up - all subjects accounted for ✵
   b) subjects lost to follow up unlikely to introduce bias - small number lost - > ____ % (select an adequate %) follow up, or description provided of those lost) ✵
   c) follow up rate < ____% (select an adequate %) and no description of those lost
   d) no statement

NEWCASTLE - OTTAWA QUALITY ASSESSMENT SCALE
COHORT STUDIES

Note: A study can be awarded a maximum of one star for each numbered item within the Selection and Outcome categories. A maximum of two stars can be given for Comparability

Selection

1) Representativeness of the exposed cohort
   a) truly representative of the average patient with PTS/NA (describe) in the community ✵
   b) somewhat representative of the average _______________ in the community ✵
   c) selected group of users eg nurses, volunteers
   d) no description of the derivation of the cohort

2) Selection of the non exposed cohort
   a) drawn from the same community as the exposed cohort ✵
   b) drawn from a different source
   c) no description of the derivation of the non exposed cohort

3) Ascertainment of exposure
   a) secure record (eg surgical records) ✵
   b) structured interview ✵
   c) written self report
   d) no description

4) Demonstration that outcome of interest was not present at start of study
   a) yes ✵
   b) no

Comparability

1) Comparability of cohorts on the basis of the design or analysis
   a) study controls for ______________ (select the most important factor) ✵
   b) study controls for any additional factor ✵ (This criteria could be modified to indicate specific control for a second important factor.)

Outcome

1) Assessment of outcome
   a) independent blind assessment ✵
   b) record linkage ✵
   c) self report
   d) no description

2) Was follow-up long enough for outcomes to occur
   a) yes (select an adequate follow up period for outcome of interest) ✵
   b) no

3) Adequacy of follow up of cohorts
   a) complete follow up - all subjects accounted for ✵
   b) subjects lost to follow up unlikely to introduce bias - small number lost - > ____ % (select an adequate %) follow up, or description provided of those lost) ✵
   c) follow up rate < ____% (select an adequate %) and no description of those lost
   d) no statement

NEWCASTLE - OTTAWA QUALITY ASSESSMENT SCALE
COHORT STUDIES

Note: A study can be awarded a maximum of one star for each numbered item within the Selection and Outcome categories. A maximum of two stars can be given for Comparability

Selection

1) Representativeness of the exposed cohort
   a) truly representative of the average patient with PTS (describe) in the community ✵
   b) somewhat representative of the average _______________ in the community ✵
   c) selected group of users eg nurses, volunteers
   d) no description of the derivation of the cohort

2) Selection of the non exposed cohort
   a) drawn from the same community as the exposed cohort ✵
   b) drawn from a different source
   c) no description of the derivation of the non exposed cohort

3) Ascertainment of exposure
   a) secure record (eg surgical records) ✵
   b) structured interview ✵
   c) written self report
   d) no description

4) Demonstration that outcome of interest was not present at start of study
   a) yes ✵
   b) no

Comparability

1) Comparability of cohorts on the basis of the design or analysis
   a) study controls for ______________ (select the most important factor) ✵
   b) study controls for any additional factor ✵ (This criteria could be modified to indicate specific control for a second important factor.)

Outcome

1) Assessment of outcome
   a) independent blind assessment ✵
   b) record linkage ✵
   c) self report
   d) no description

2) Was follow-up long enough for outcomes to occur
   a) yes (select an adequate follow up period for outcome of interest) ✵
   b) no

3) Adequacy of follow up of cohorts
   a) complete follow up - all subjects accounted for ✵
   b) subjects lost to follow up unlikely to introduce bias - small number lost - > ____ % (select an adequate %) follow up, or description provided of those lost) ✵
   c) follow up rate < ____% (select an adequate %) and no description of those lost
   d) no statement

Newcastle-Ottawa Quality Assessment Form for Cohort Studies

Note: A study can be given a maximum of one star for each numbered item within the Selection and Outcome categories. A maximum of two stars can be given for Comparability.

Selection

1) Representativeness of the exposed cohort
   a) Truly representative ***(one star)***
   b) Somewhat representative ***(one star)***
   c) Selected group
   d) No description of the derivation of the cohort

2) Selection of the non-exposed cohort
   a) Drawn from the same community as the exposed cohort ***(one star)***
   b) Drawn from a different source
   c) No description of the derivation of the non exposed cohort

3) Ascertainment of exposure
   a) Secure record (e.g., surgical record) ***(one star)***
   b) Structured interview ***(one star)***
   c) Written self report
   d) No description
   e) Other

4) Demonstration that outcome of interest was not present at start of study
   a) Yes ***(one star)***
   b) No

Comparability

1) Comparability of cohorts on the basis of the design or analysis controlled for confounders
   a) The study controls for age, sex and marital status ***(one star)***
   b) Study controls for other factors (list) ________________________________ ***(one star)***
   c) Cohorts are not comparable on the basis of the design or analysis controlled for confounders

Outcome

1) Assessment of outcome
   a) Independent blind assessment ***(one star)***
   b) Record linkage ***(one star)***
   c) Self report
   d) No description
   e) Other

2) Was follow-up long enough for outcomes to occur
   a) Yes ***(one star)***
   b) No

Indicate the median duration of follow-up and a brief rationale for the assessment above:____________________

3) Adequacy of follow-up of cohorts
   a) Complete follow up- all subject accounted for ***(one star)***
   b) Subjects lost to follow up unlikely to introduce bias- number lost less than or equal to 20% or description of those lost suggested no different from those followed. ***(one star)***
   c) Follow up rate less than 80% and no description of those lost
   d) No statement

Thresholds for converting the Newcastle-Ottawa scales to AHRQ standards (good, fair, and poor):

Good quality: 3 or 4 stars in selection domain AND 1 or 2 stars in comparability domain AND 2 or 3 stars in outcome/exposure domain

Fair quality: 2 stars in selection domain AND 1 or 2 stars in comparability domain AND 2 or 3 stars in outcome/exposure domain

Poor quality: 0 or 1 star in selection domain OR 0 stars in comparability domain OR 0 or 1 stars in outcome/exposure domain
